# Magnetic resonance imaging in traumatic hip subluxation

**DOI:** 10.4103/0019-5413.80048

**Published:** 2011

**Authors:** David C Flanigan, Arthur A De Smet, Ben Graf

**Affiliations:** Department of Orthopedics, The Ohio State University, Columbus, Ohio, USA; 1 Department of Orthopedic Surgery and Rehabilitation, Division of Sports Medicine, University of Wisconsin Hospital and Clinics, Madison, Wisconsin, USA

**Keywords:** Hip subluxation, magnetic resonance imaging, sports injuries

## Abstract

Athletic traumatic hip subluxations are rare. Classic radiographic features have been well described. This case highlights the potential pitfalls of immediate magnetic resonance imaging. Femoral head contusions and acetabular rim fractures are common associated findings usually apparent with magnetic resonance imaging (MRI). However, in this case an MRI done 3 hours post injury failed to show any edema in either location, making the appearance of these findings on subsequent MRIs difficult to interpret. An acute MRI more than 48 hours post injury may have been more helpful.

## INTRODUCTION

Traumatic hip subluxations and dislocations are rare athletic injuries. These injuries can have severe long-term consequences, most notably avascular necrosis of the hip. Most of our knowledge in treating these injuries has come from case reports and case series.^1^–^3^ This case report highlights the role of radiographic imaging in the evaluation and management of these cases.

## CASE REPORT

A 20-year-old collegiate football lineman was blocking during practice, when he was forced from his left side over an extended right leg, creating an internal rotation and adduction moment of the hip. He felt a ‘sliding and crunching sensation’ as he was driven over his right hip. He fell to the ground, and immediately had pain in his right hip. Initial evaluation on the field by the training staff and physician revealed that the hip had full motion, but there was pain with rotation of hip and inability of the athlete to bear weight. The patient was transferred to the emergency room for radiographs and further evaluation.

**Figure 1:**
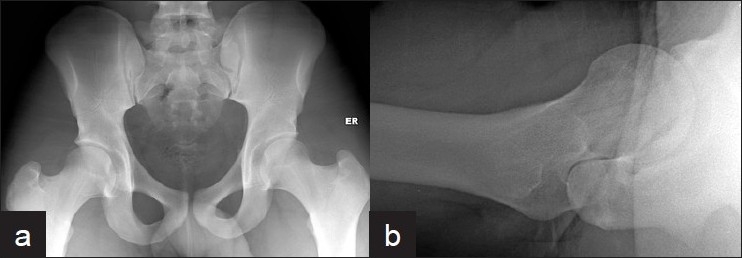
(a) Anteroposterior view of pelvis and (b) cross table lateral view of the right hip several hours after injury showing normal radiograph

**Figure 2:**
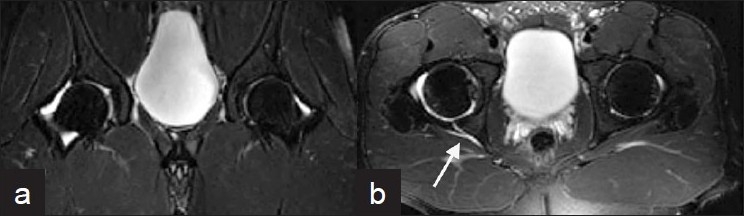
MRI performed three hours after injury. (a) Coronal fast spin echo T2-weighted image with fat saturation shows a small right hip effusion and no marrow abnormality. (b) Axial fast spin echo T2- weighted image with fat saturation shows a right posterior labral tear (arrow) with fluid dissecting posterior to the acetabulum. (Images obtained on a 1.5T General Electric MR scanner)

In the emergency room, the athlete complained of further pain in the hip which was increased with hip rotation. Initial radiographs consisted of pelvis anteroposterior view and lateral view of right hip [Figure 1]. The radiographs did not reveal any fracture or dislocation. With his persistent symptoms and continued pain with rotation, magnetic resonance imaging (MRI) was obtained to rule out an occult fracture within three hours of the initial injury. The MRI revealed a small hemarthrosis and a posterior labral tear, but there was no iliofemoral ligament tear or bone edema in the acetabulum or femoral head [Figure 2]. The patient was discharged with instructions to not bear weight on the right hip, and use crutches.

The athlete was re-evaluated the next day and found to have continued tenderness with internal rotation and with stressing of the posterior capsule and labrum. He continued to feel a ‘sliding sensation as if (his) hip was popping out,’ although this was not reproducible on examination. A diagnosis of traumatic hip subluxation was made based on his original injury, continued feelings of slipping, and his examination findings. The athlete was placed on hip precautions (patient was instructed to limit internal rotation of the hip, limit adduction of hip, sleeping on back with pillows between the leg) and continued on non-weight bearing, with crutches, for six weeks.

**Figure 3:**
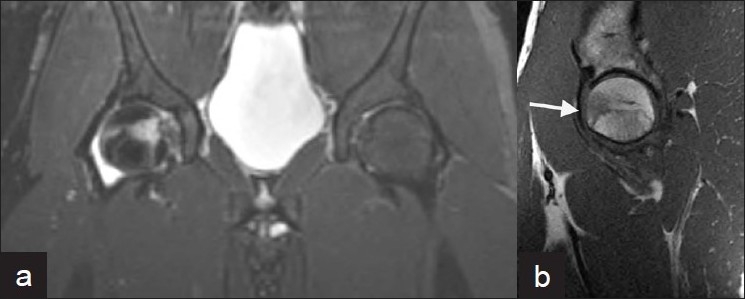
MRI six weeks after injury. The coronal fast spin echo T2- weighted image with fat saturation (a) and the sagittal T1-weighted (b) image show a new abnormality in the anterior aspect of the right femoral head, which is with low signal intensity on T1 weighted and high signal intensity on T2 weighted. (Images obtained on a 1.5T General Electric MR scanner)

Over the course of this time, the athlete’s symptoms and capsular signs resolved. Radiographs (AP pelvis, AP and lateral hip films) at six weeks were unremarkable. An MRI was obtained to search for signs of avascular necrosis (AVN) and to determine the safety of a possible clearance for return-to-play. An increased signal was observed in a well-localized area in the superior anterior aspect of the femoral head near the fovea on the T2-weighted images [Figure 3]. The location was atypical for AVN, but the lack of edema in the same area immediately post injury made these changes worrisome. The differential diagnosis was atypical AVN (by location) or an atypical femoral head bone bruise (by the lack of edema on the initial MRI). On account of the uncertainty of diagnosis the patient was continued on toe touch weight bearing for a further six weeks, but allowed gentle range of motion exercises with the trainers. The patient reported compliance with all physician instructions.

**Figure 4:**
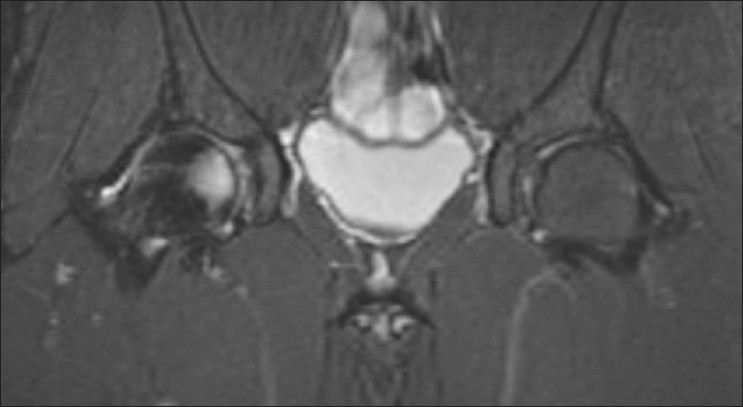
MRI three months after injury. The coronal fast spin echo T2-weighted image with a fat saturation image shows slight increase in size and signal intensity of the right femoral head abnormality. (Images obtained on a 1.5T General Electric MR scanner)

**Figure 5:**
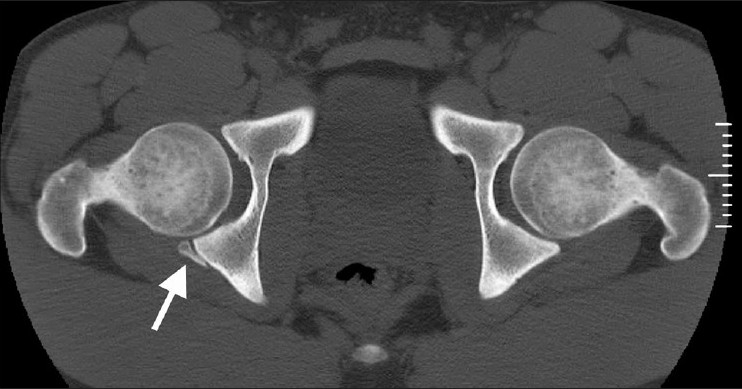
CT three months after injury shows a posterior lip right acetabular fracture with bridging bone at the posterior margin of the fragment

An MRI was obtained three months after injury [Figure 4]. The lesion on T2 weighted images was still present in the same location and had increased in size and signal intensity. A CT scan was performed to better delineate this lesion and revealed a posterior lip acetabular fracture [Figure 5].

The patient remained on restricted activities, but was allowed to start range of motion activities and non-impact conditioning under the supervision of the athletic trainers.

**Figure 6:**
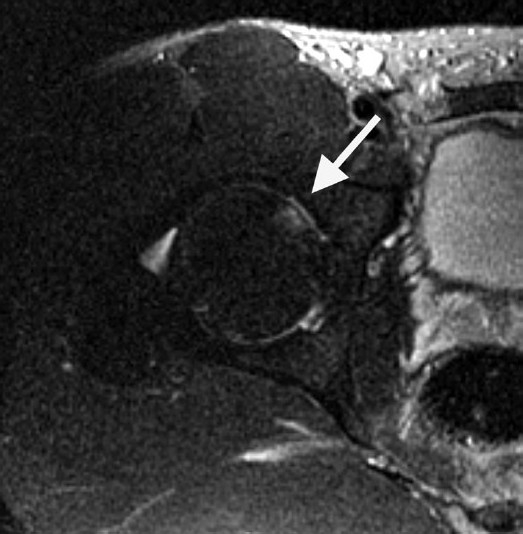
MRI 10 months after injury was normal except for a tiny focus of residual edema in the anterior aspect of the right femoral head

Subsequent MRIs at five and 10 months post injury revealed progressive improvement with almost complete resolution of the abnormal signal intensity [Figure 6]. The athlete progressed with training under supervision after his five month MR, and continued, within two months, to unsupervised training for football without any episodes of instability, clicking or pain. He returned to competitive football the following season, 12 months after injury without incident. The final diagnosis was hip subluxation with bone bruise of the femoral head that was not seen on the acute MRI. The subject was informed that data concerning the case would be submitted for publication.

## DISCUSSION

Traumatic hip subluxations and dislocations are rare injuries for athletes. The most comprehensive series of this athletic injury and guide to management has been elaborated by Moorman *et al*.^2^ In this series, the mechanism, physical, and radiographic findings of eight American football players who sustained traumatic, episodic hip subluxations were reviewed. In six of eight, the mechanism was a fall on a flexed knee with the hip in a flexed and adducted position, similar to the position of the hip during traumatic hip dislocation resulting from a motor vehicle collision. One athlete had a mechanism similar to our reported case. The radiographic and imaging features that were identified in this series were: posterior acetabular lip fracture, hemarthrosis, and disruption of the iliofemoral ligament.^2^

Typically, the initial AP and lateral radiographs are negative. Judet views are recommended to identify the posterior acetabular lip fracture that was seen in all eight patients of Moorman *et al*. series.^2^ This series of radiographs were not obtained in our patient because of the lack of acetabular rim edema, which erroneously suggested that a rim fracture was unlikely. In contrast, all of Moorman *et al*.’s patients who had an initial MRI within the first week after injury had edema in the region of the acetabular lip fracture. This case suggests that Judet films should be obtained in all cases, regardless of the MRI findings.

In the Moorman *et al*.^2^ series, seven of eight athletes had an initial MRI within the first week after the injury, and in all seven cases the femoral head appeared viable. One of these seven athletes developed osteonecrosis, which was identified by the six-week, follow-up MRI.^2^ In our study, the initial MRI was negative for any abnormality in the femoral head, but at six weeks the imaging revealed edema in the fovea of the femoral head and the acetabulum. Our expectation was that a bone bruise would have demonstrated edema on acute MRI. Although the lesion did not have the typical characteristics of osteonecrosis with regard to location, subchondral collapse or the double line sign,^4^,^5^ we did not feel comfortable eliminating AVN as a diagnostic possibility, until the lesion resolved seven to nine months post injury. Had the initial MRI revealed a bone bruise, then this case would have much less diagnostic dilemma.

The risk of osteonecrosis was the greatest concern with this athlete. With a brief subluxation the risk of AVN was considered to be low. Typically, the anterosuperior aspect of the femoral head would have involved in osteonecrosis and not the anterior aspect as present on these MRIs. Although AVN could spontaneously resolve by having less edema on T2W MRI over time, but typically, persistent changes and findings were seen on an MRI. In this case, the location of the lesion was atypical for AVN and the MR findings fully resolved with no residual effect. This was why the authors believed it was consistent with a bone bruise, as was also suggested by Laorr and Blankenbarker’s reports.^6^,^7^ Moorman’s series did not describe any changes to the femoral head outside of the case of AVN; and these findings persisted until requirement of a total hip replacement.^2^ Furthermore, the location of AVN was typical in this case. The details of all the MRI findings in the femoral head were initially lacking in this study, and therefore, it was difficult to draw comparisons to this case.

Magnetic resonance imaging has been extremely useful in diagnosing injuries and their associated findings. Anterior cruciate ligament injuries and hip dislocations have associated bone contusions often seen in the acute setting. Bone contusions classically have geographic, non-linear areas of signal loss on T1-weighted images and high signal intensity on T2-weighted images. In acute anterior cruciate ligament injuries, bone bruises in the lateral compartment are common after an acute injury and usually resolve within three months, but may persist longer.^8^–^13^ An MRI can also document femoral head contusions caused by dislocations. In the study by Laorr *et al*., femoral head contusions were found in 14 of 18 patients with hip dislocations who had an MRI two to 35 days after injury (mean 13 days).^6^ What is not known is the absolute time from injury, required for these associated findings in the hip to become present on the MRI. A swine study by Blankenbarker *et al*. suggests that bone bruising may be evident in MRI examination as early as one hour before trauma, but can take as long as 30 hours.^7^ The present case suggests that it may be necessary to have a longer time gap (48 – 72 hours) after injury, to accurately identify the injury and the associated findings.

How can one reconcile a clinical course and outcome consistent with a femoral head bone bruise and the lack of femoral head and acetabular rim edema on an acute MRI? One unusual feature of this case was that the MRI was performed within three hours of the injury. It seems likely that this interval was so short that the characteristic changes of edema due to the bone bruise and rim fracture did not have time to develop. It may be necessary to have a longer time gap (48 – 72 hours) after injury, to accurately identify the injury and associated findings.

This case highlights the importance of a complete radiographic workup for a potential traumatic hip subluxation. Judet views are recommended in all cases, to identify the acetabular lip fracture, as recommended in the literature.^2^ An acute MRI can assist in making the diagnosis of hip subluxation, but probably should be delayed 48 to 72 hours, so that bone bruises can be accurately diagnosed. Follow-up magnetic resonance imaging at six weeks is necessary to rule out osteonecrosis prior to clearance for return to play. A bone contusion may be an associated finding with this injury and can take months to resolve. An MRI done within hours of injury can be difficult to interpret.
